# A Data-informed Public Health Policy-Makers Platform

**DOI:** 10.3390/ijerph17093271

**Published:** 2020-05-07

**Authors:** Dario Brdarić, Senka Samardžić, Ivana Mihin Huskić, Giorgos Dritsakis, Jadran Sessa, Mariola Śliwińska-Kowalska, Małgorzata Pawlaczyk-Łuszczyńska, Ioannis Basdekis, George Spanoudakis

**Affiliations:** 1 Department for Disinfection, Disinsection and Deratization, Institute of Public Health for the Osijek Baranya County, 31000 Osijek, Croatia; dario.brdaric@gmail.com (D.B.); senka.002@gmail.com (S.S.); mihin.ivana@gmail.com (I.M.H.); 2 Department of Public Health, Humanities and Social Sciences in Biomedicine, Faculty of Dental Medicine and Health, J. J. Strossmayer University of Osijek, 31000 Osijek, Croatia; 3 Ear Institute, University College London, London WC1E 6BT, UK; g.dritsakis@ucl.ac.uk; 4 Dipartimento di Informatica, Università degli Studi di Milano, 20133 Milano, Italy; 5 Department of Physical Hazard, Nofer Institute of Occupational Medicine, 91-348 Łódź, Poland; msliwinska@imp.lodz.pl (M.Ś.-K.); malgorzata.pawlaczyk@imp.lodz.pl (M.P.-Ł.); 6 Department of Computer Science, City University of London, London EC1V 0HB, UK; ioannis.basdekis@city.ac.uk (I.B.); g.e.spanoudakis@city.ac.uk (G.S.)

**Keywords:** policymaking, big data analytics, health

## Abstract

Hearing loss is a disease exhibiting a growing trend due to a number of factors, including but not limited to the mundane exposure to the noise and ever-increasing size of the older population. In the framework of a public health policymaking process, modeling of the hearing loss disease based on data is a key factor in alleviating the issues related to the disease and in issuing effective public health policies. First, the paper describes the steps of the data-driven policymaking process. Afterward, a scenario along with the part of the proposed platform responsible for supporting policymaking are presented. With the aim of demonstrating the capabilities and usability of the platform for the policy-makers, some initial results of preliminary analytics are presented in the framework of a policy-making process. Ultimately, the utility of the approach is validated throughout the results of the survey which was presented to the health system policy-makers involved in the policy development process in Croatia.

## 1. Introduction

According to the Polityka Insight [1], “eHealth is the application of information and communications technologies across the whole range of functions that affect the health sector. This broad definition encompasses a variety of digital applications, processes, and platforms, including electronic health record systems, telehealth (remote medical consultation), smartphone apps, remote monitoring devices and biosensors, computer algorithms, and analytical tools to inform decision making”. The development of health policies can lead to ineffective and/or inefficient health systems posing a growing threat to a nations’ public health and economic stability, due to a lack of use or consultation that is based on the best available research evidence. According to Lavis et al. [2], the ultimate goal of a health system is to promote community health in an equitable manner and, as such, evidence is required to enable policy-makers to assess the effectiveness of health policy in question. Consequently, ineffectiveness could be minimized by incorporating state-of-the-art information technologies into the process of policy-making, i.e., using evidence-based approaches suggested by Ellen et al. [3]

Israeli researches found differences in the perceptions of knowledge producers and knowledge users on factors hindering the implementation of health policy and systems research, the accessibility of evidence to policy-makers, evidence credibility, and groups/factors influencing health policymaking [4]. Furthermore, in the United States of America, health models are simulated when working on health reforms, which usually results in the creation of accurate estimates for specific health policies, as described in the work by Glied et al. [5] Different public health issues are often dynamic and complex. Several approaches can be used in the policy modeling of chronic disease prevention, including specific factors as an ecological approach, disease outcomes, health and risk behaviors, environmental factors, health-related resources, and delivery systems [6]. Mathematical modeling is a key issue when dealing with disease prevention. Metcalf et al. [7] described six challenges between modeling and public health: C1. Communicating the limits of modeling, C2. Maintaining the value of models in the face of long-time horizons, C3. Usefully deploying modeling in the context of ‘black swans’, C4. Integrating modelers and model-building into the policy process, C5. Economic analysis and decision support, and C6. Creating a cycle where results inform decisions and vice versa. Models are described and developed as computational versions of laboratory experiments and using available data. That kind of approach can be used when searching for answers on policy questions. The final results of policy modeling should be transparent and easy to replicate [8]. Phases of the policymaking process are described by Dunn [9] as cycles of agenda setting, policy formulation, policy adoption, policy implementation, policy assessment, policy adaptation, policy succession, and policy termination.

There is a number of projects and papers that try to address the policymaking challenges. The research project entitled ‘evidence-based management of hearing impairments: public health policymaking based on fusing big data analytics and simulation’ (EVOTION) [10] aims to build an evidence base for the formulation of public health policies related to the prevention, early diagnosis, long-term treatment, and rehabilitation of hearing loss (HL), as well as the detection and prevention of cognitive decline and the socio-economic inclusion of individuals with HL. Its objective is to enable and support more holistic management of HL (EVidenced based management of hearing impairments: Public health pOlicy making based on fusing big data analytics and simulation (EVOTION). Available online: http://h2020evotion.eu/). In the other words, even though it has potential uses in the other health sectors, EVOTION is specifically tailored for addressing HL-related issues and policy making. HL is a disease with the increasing prevalence, and it will increase due to increasing exposure to noise and an increase in the aging population. Anisetti et al. [11,12,13] use a big data platform to identify the behavior of interest and deontic logic in order to design a data-driven model for policymaking that can be updated over time. Prasinos et al. [14] propose a model-based approach presenting a platform for policy-makers in the context of HL related policies. This platform is based on high-level language for the specification of evidence-based health policy and decision-making models based on big data analytics. In this paper, we propose a platform to support data-informed policymaking.

According to Innvaer et al. [15], “the empirical basis for theories and common wisdom regarding how to improve appropriate use of research evidence in policy decisions is unclear. One source of empirical evidence is interview studies with policy-makers”. In this paper, we first describe the data-informed policymaking process. We then present a platform capable to support such a process and address the above challenges (C1–C6) and then present some preliminary walkthroughs of its usage. In addition, as a way to evaluate the value of the approach from a policymaking perspective, we present a survey to find out what experts, who are involved in creating public health policies and making decisions, think about e-health and the platform we proposed.

## 2. The Data-informed Policymaking Platform

Our policymaking platform is an analytics-as-a-service platform based on the TOREADOR [16] methodology and fine-tuned for the needs of policy-makers. It allows the use of fully parametric predefined analytics (analytics workflows) to be tailored to specific needs, as well as building analytics from scratch, combining analytics methods (analytics tasks) into a workflow following the approach in the work of Anisetti et al [11].

In this section, we first describe the data-informed policymaking process to foreground our platform supporting this process.

### 2.1. The Policymaking Process

In the framework of defining a health policy, policy-makers are normally assisted by domain experts that rely on previous scientifically validated results to be used to support a specific policy decision. Unfortunately, scientifically validated results are not always available/suitable for all the situations. For instance, in emergency situations, as it happened with COVID-19 pandemic, many collateral aspects including health and economic aspects have to be taken into consideration. When well-established literature or well known best practices are not available the policymaking process is much more complex and normally less effective requiring time and multiple refinement iterations. This paper presents a platform that can assist policy-makers to retrieve the evidence they need in order to take an effective and rapid decision on specific policy action. This platform allows access to a set of analytical capabilities that, when associated with a data lake where data relevant for the domain of the policy is continuously fed, can allow for the retrieval of evidence needed for a policy release on a specific domain. This means that a simulation can be run on the already existing data, which can, as a result, provide insights about how a certain policy recommendation can impact the study population.

In this paper, we assume a scenario where all the relevant data needed for a specific domain is available (e.g., open data or validated medical longitudinal study).

We also assume as normal in case of policymaking that the policy-maker is assisted by a domain expert while setting up a policy. In our case, this domain expert is also a data scientist capable to select among the available analytics offered by the platform or to compose them into new ones.

**Figure 1:**
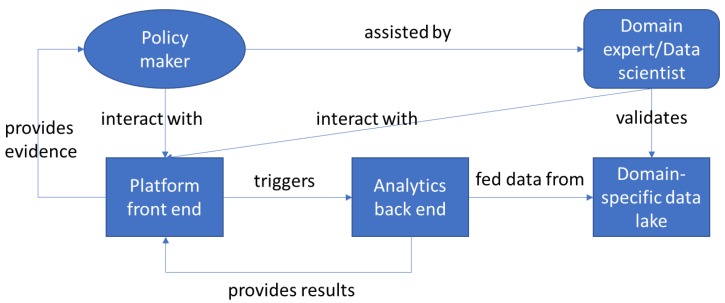
Data-Informed Policymaking Methodology.

Figure 1 shows the methodological view of our data-informed policymaking process. In the framework of retrieving evidence for defining a required policy, policy-maker can interact with the platform front end with the assistance of a domain expert. The interaction is aimed to trigger analytics on a data lake that is relevant for the policy (e.g., clinical data form COVID patients). When executed the analytics provides results that have to be aggregated into meaningful evidence to be provided to the policy-makers. The platform can also be used by a domain expert to validate the data in the data lake (e.g., statistical validity, coverage, etc.). Normally, validation is the preliminary step in order to ensure that the dataset is informative enough to derive meaningful results.

While interacting with the platform front end the policy-maker fulfills a data-informed policymaking process that according to Anisetti et al. [11,12] is based on a sequence of steps as follows.

Situation Analysis: the preliminary stage where the policy-makers decided how to proceed and draft the policy to be refined and validated in the following stages.

Action Plan: Traditionally this consists of selecting patients for a trial. In the scenario of policymaking driven by data, this is equivalent to selecting data from the available set on one side and enrolling additional patients on the other (i.e., selecting patients).

Implementation Evaluation and Monitoring: The policy is released as a recommendation and later moved to prescription in case of successful verification of the validity. This means that the data are still being collected from the set of enrolled patients in order to see the impact of the policy. Monitoring could be a long-term process whereby the policy is monitored in order to see if some fine-tuning is required or not.

Depending on the outcome of this stage the policymaking process can be also partially or completely re-executed from situation analysis or from the action plan stage. Each stage of the data-informed policymaking process requires different types of analysis.

**Figure 2:**
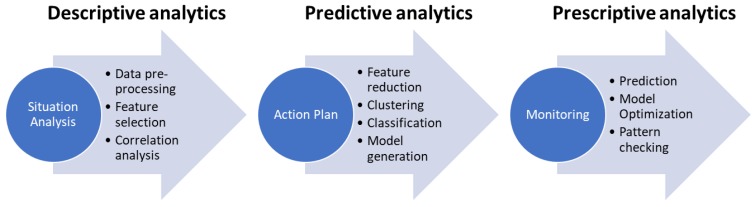
Data-Informed Policymaking process.

Figure 2 shows our data-informed policymaking process connected to the analytical activities that are requested to support each of the policy stages.

During the situation analysis step, the policy-makers can: (i) analyze what has been done in literature for the aspects they want to take care of and for instance identify which features have been considered in similar situations and why, and (ii) do some preliminary analysis on the distribution and correlations of these important features to envision how to proceed further. They can also add other features that have not been considered in literature in order to see if they are relevant or not. At this stage, the analysis is of a descriptive type, like aggregation functions and simple statistical analysis.

During the action plan step, the policy-maker can: (i) given the preliminary results set up the predictive analytics that will support the policy, (ii) verify with the preliminary collected set of data and the historical data the validity of the prediction, and (iii) define the policy. At this stage, the analysis is of the predictive type, like machine learning approaches for classification, prediction, and pattern identification.

During the implementation evaluation and monitoring step, the effect of the policy will be monitored using simulation, optimization and prediction capabilities. This stage of analysis is of a prescriptive type. Moreover, the focus of this stage lies in performing fine-tuning and optimization, as well as for deciding whether the discrepancy between what is predicted and what actually occurred requires further refinement at the action plan or situation analysis stages.

### 2.2. The Platform

Our platform supports the methodology in Figure 1 and the above policymaking process in Figure 2. It is constituted by a number of components.

**Figure 3:**
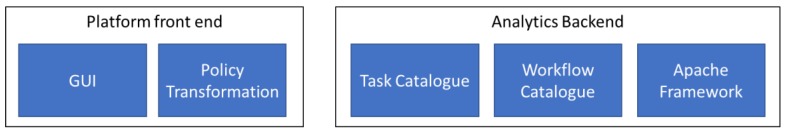
Platform Components.

Figure 3 shows the principal platform components. More in detail the Analytics backend is an evolution of the big data engine described in the work of Rathore et al. [17] that builds on Apache Framework, i.e., Hadoop Distributed File System (HDFS), Hadoop, and Spark just to name the few, enabling processing of a large amount of data. The need for such a big data platform, considering the 5v definition, is due to the nature of the policymaking analytics that spans over a population of a region or entire country (i.e., volume). It includes different sources and formats (i.e., variety), concerning in most of the cases data trustworthiness and sensibility (i.e., value, veracity). Occasionally, when the policy is in the monitoring stage, it is able to process incoming new data from the policy application in order to monitor the effectiveness (i.e., velocity).

In order to support the policymaking process the Apache platform is enriched with two catalogs that offer analytical capabilities to policy-makers:

- Analytics task catalog: A catalog of analytics tasks offered by the platform. For instance, statistical analyses, feature selections, but also K-means clustering, KNN model, and prediction just to name a few. The analytics tasks are designed to be composed into workflows and are based on the available mllib library for Spark2 and ad hoc processing tasks.
- Analytics workflows catalog: A catalog of workflows that can be triggered to perform the analysis. The workflows are orchestrated with Oozie.

The platform front-end provides a graphical interface for the policy-maker to: (i) select between the available workflows, choosing parameters, like the specific data source to be used, or (ii) build new ones composing available tasks into workflows.

While composing a new workflow the policy-maker is guided by the platform in order to produce a valid workflow by design. The guide is constituted by a set of template workflows to be fed with analytics tasks. Some examples of template workflows include modeling workflows, which can be used to generate a machine learning model via training and prediction template which can be used to perform a prediction based on a specific model. There are also preprocessing template workflows that can be used for feature selection, data cleaning, normalizations, etc. The template approach is much more generic than the parametrized workflows since it allows a policy-maker to choose between a number of different analytical algorithms available in the task catalog and combine them in case of necessity or to compare them.

For each step of the workflow creation, the relevant available tasks for a specific template are shown to the policy-makers as well as all the required parameters to be decided.

In order to support this process, the front end is composed of the following sub-components

- GUI: The graphical interface where the policy-maker can easily select and parametrize available workflows or build a new one with a wizard-like assisted interface.
- Transformation service: Transforms the workflows generated at interface level into executable ones building the required glue into Oozie workflows. It first checks that it is valid and then transform it.

Our platform covers the requirements listed in the introduction (C1–C6).

The process implemented by the platform is mainly driven by the data as well as by the analytics model and required policies for data processing. This allows the comparison and selection of the most representative model to be used in relation to a specific policy (C1,C4). The results of the policy model executions are saved in the database and maintained for future use and comparison (C2). The platform is capable of executing predictions in a timely manner and supporting predictions in the emergency, and not expected situation (C3). Furthermore, the platform can be used as a means to support the decision of the policy-makers providing evidence and can be extended to take care of the economics connected (C5). Finally, the platform also fosters a refinement cycle where results can be used for additional analysis until it converges to a policy intended for release (C6). The policy-maker can schedule the execution of each workflow and be notified when the execution completed.

Policy-makers can also see the list of all previously executed workflows along with their execution status and, in case of necessity, they can re-execute them. For each executed workflow the domain expert can see more advanced details like the graphical representation of the workflows, including their building blocks (tasks), as well as logs, intermediate results, and specified configurations for each of the building blocks.

Once the desired workflow has been successfully executed, it is saved to the database (i.e., HDFS). Afterward, a policy-maker can choose to use the saved data in the framework of another analysis or visualize the results through the means of front end GUI. Furthermore, a policy-maker can select the desired visualization option, such as a histogram or line chart and a resulting dataset, and fill the required input parameters in order to get a better perception of the produced results.

## 3. The Hearing Loss Scenario

In this paper, we consider a hearing loss (HL) policy scenario of the h2020 EVOTION project where a trial with a significant population of HL patients has been selected and equipped with hearing aids (HA) devices in order to study their subjective satisfaction as well as significant changes to the HA usage data been produced, in the context of the policymaking process. More specifically, the trial includes around 1000 subjects fitted with HA devices and monitored for one year. The dataset contains subject data including age, education level, and a set of results of medical tests carried out during the fitting visits and the periodical follow-up visits. The dataset also consists of more advanced parameters related to the degree of hearing loss in each ear, such as the combined hearing loss level for both ears and measure of the MOCA (Montreal Cognitive Assessment) test, as well as details regarding HA usage and the relative parameters (average usage per day for instance).

For the sake of this paper, since we are just demonstrating the platform utility and not scientific relevance of the analysis carried out, the data used was synthetically generated starting from the EVOTION trial dataset following the approach of Christensen et al. [18] which permits the preservation of the privacy and maintenance of the statistical significance. The policy’s goal that we are considering is to minimize/lower the dropout rate, i.e., number of the patients who withdrew from the study prematurely, from the clinical study predicting dropping out factors with the aim of effective intervention or as a way of planning a better subject selection to minimize the number of dropouts. The dropout from a study is physiological, and in many situations may cause a number of issues related to the effectiveness of the study. There is a set of potential causes that can lead patients to drop out of the study. These factors have to be thoroughly scrutinized in order to elucidate the factors which could lead to the deterioration of the overall study quality and which can help in the future prediction of patients or groups of patients that are under the risk of leaving the study prematurely.

This scenario poses challenges from a medical and technical perspective, since the HL pathologies deeply depend on the subject behavior and HL has been shown to have a significant societal impact. From the policymaking point of view is therefore very representative.

## 4. Preliminary Evaluation

In this section, we first present some preliminary policymaking results applied to the scenario in Section 3, describing a walkthrough example. Secondly, to evaluate the utility of the approach from the policy-maker perspective, we first presented the platform to the policy-makers and then surveyed.

### 4.1. Policymaking Platform: a Walkthrough Example

The following walkthrough describes the process of policymaking with the goal of reducing the dropout rate (see the scenario described in Section 3). Even though the platform supports the entire process, for the sake of conciseness, we concentrated on the situation analysis and action plan only, leaving the monitoring stage for future works.

#### 4.1.1. Situation Analysis

Let us consider the situation analysis step of the policymaking process, a policy-maker first tries to inspect the current literature about the factors affecting the dropout rate in the context of HL. Using our platform, the policy-maker can see if the features that come from the literature are available in the data lake connected to the platform or if they can be calculated starting from what is available. For instance, if one feature that is relevant in the literature related to the scenario of this walkthrough is the total usage of the HA device per month, but the dataset contains just the timestamp where the HA device has been turned on and off by a given subject, there is the need to compute the usage time as the sum of these periods for each subject for a month. The platform supports policy-makers in doing these preliminary analyses offering all the pre-processing capabilities requested. The policy-maker can combine analytics tasks into workflows to perform the data pre-processing.

Having done the pre-processing workflow, the policy-makers can use the computed total HA usage (6262105 records in our case for all the 1000 subject of the trial) to carry out two critical trends analyses that have been shown and that are correlated to the dropout rate of the patients, namely:

- Average HA usage among the entire population grouped by the participants’ age for each month since the fitting date.
- Average HA usage among the entire population grouped by the participants’ education level age for each month since the fitting date.

In the first group the participants have been divided into several groups according to their age and overall distribution in each group, including (i) participants younger than 35 years; (ii) participants between 35 and 45 years old; (iii) participants between 45 and 55 years old; (iv) participants between 55 and 65 years old; (v) participants between 65 and 75 years old; (vi) participants older than 75 years.

Similarly, participants are divided into three groups according to their education level, namely:

- Participants having low education level (participants that have spent eight or fewer years in education, i.e., equivalent to elementary school).
- Participants having medium education level (participants that have spent between 9 and 14 years in education, i.e., equivalent to the secondary education).
- Participants having high education levels (participants that have spent 14 or more years in education, i.e., equivalent to the University degree).

**Figure 4:**
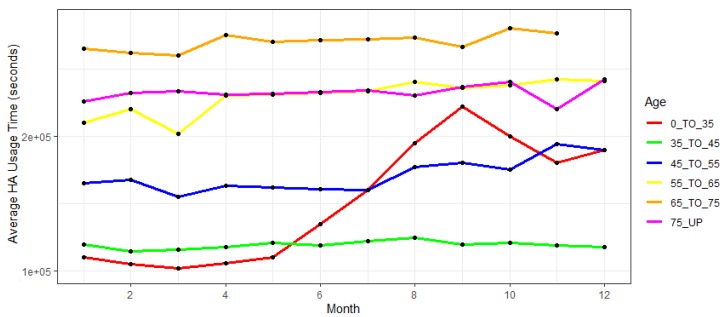
Average HA usage per age group per month.

**Figure 5:**
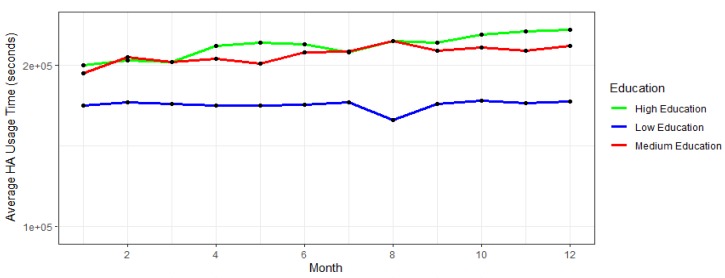
Average HA usage per age group per month.

The results of analyses of these two trends are shown in Figure 4 and Figure 5, which can be seen below.

As it can be observed from the given Figure 4, the results indicate that the older participants, aged from 65 to 75 years, have been using HA device the most consistently throughout investigated time, followed by the group of patients aged 75 years or more and 55 to 65 years old. On the other hand, younger (below 35 years old) and middle-aged participants (45 to 55 years old) show a similar, significantly lower trend of HA usage. Participants aged from 35 to 45 years old tend to be the riskiest group when it comes to the odds of dropping out, being constantly at the bottom of average HA usage. Moreover, it can be indicated that the participants belonging to this age group exhibit a gradual decrease in HA usage during the time.

Figure 5 demonstrates the average HA usage per education level group during the course of the time. Highly educated participants show both the best progress, i.e., constant growth of HA usage, except for the last month, as well as the highest average HA usage over the examined period. The second group of the participants, i.e., participants having a medium level of education exhibit the highest HA usage in their starting month; however, as time passes slower growth and stagnation can be indicated in their HA usage. Lastly, participants having lower education levels exhibit a lot of oscillations in their average HA usage. Thus, this group of participants can be considered to be most likely under the risk of dropping out from the further study.

**Figure 6:**
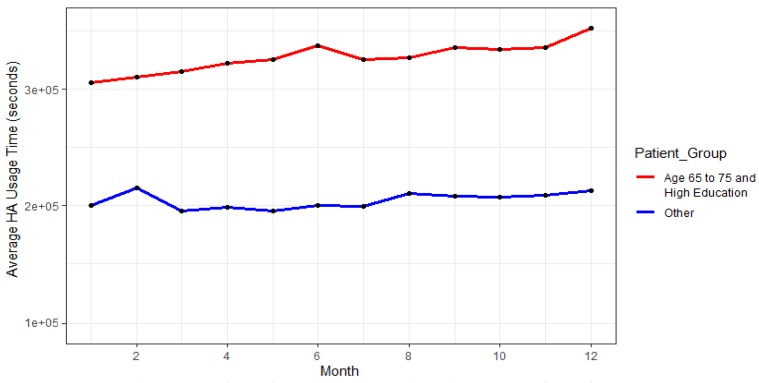
Comparison of average HA usage of the highly educated participants aged 65 to 75.

Results of the showcased analyses suggest that highly educated patients, aged 65 to 75 years are the safest group for the study. Figure 6 indicates a comparison of the average HA usage of this group compared to the average HA usage of the remaining participants.

As can be observed from the given figure, the very positive trend of HA usage and overall high average usage time can be observed for this given group.

The performed analyses reveal some of the essential trends that could be utilized by policy-makers in order to aid them in deciding on which groups of participants they should focus on future studies. These analyses concluded the situation analysis stage of the policymaking process. They give ideas on how to proceed with the other stages for which our platform provides prediction and simulation capabilities. In this case, for instance, it seems that old and highly educated patients do not drop out on average.

This evidence will be used in the next stages to tune the analysis and decide the dropout prediction analytics to be used and the features to be considered.

#### 4.1.2. Action Plan Stage

With respect to the action plan, based on the preliminary results (in terms of features and corresponding correlations), the policy-makers can select the most suitable analysis approach for achieving the policy goal among the available approaches suggested by the platform. In the case of our scenario, the goal is to predict dropout in order to see what is impacting it and release a policy capable of selectively preventing the dropout. In this walkthrough, we show how the policy-maker adopts a trial and error approach in order to find suitable analytics for its goal.

Given the analysis on the previous policymaking stage, the policy-makers selects the following features available in the data set of our scenario that show a certain degree of correlation with the dropout: AVG_HA_USAGE (average hearing aids usage), TOTAL_HA_USAGE (total hearing aids usage), VARIANCE_HA_USAGE (Variance Hearing Aids Usage), TOTAL_SCORE (MOCA MENTAL Score), AGE (patient’s age), HI_DEGREE_CURRHL_L (degree of hearing loss in the left ear), HI_DEGREE_CURRHL_R (degree of hearing loss in the right ear), HEARING_LOSS_SEVERITY (combined hearing loss level for both ears), and EDUCATION_LEVEL (education level).

In addition, since the entire dataset was labeled for the dropout, the policy-maker also selects the classification label called DROPOUT(1: dropout, 0: non-dropout) as the label, for the list of known dropout patients, as well as for the other patients which are still in the study. In order to reduce the bias and deal with the class imbalance, the number of records containing non-dropout users was reduced to the same number (134 records for each label) as the number of available patients marked as dropouts.

For demonstrating prediction capabilities of our platform, three different classification algorithms, namely decision tree classification, logistic regression and support vector machines (SVM) were selected. Afterward, the previously selected dataset was randomly split into training and testing datasets by using three different ratios: 70:30 (i.e., 70% training and 30% testing), 60:40 (i.e., 60% training and 40% testing), and 50:50 (i.e., 50% training and 50% testing). Furthermore, for the purpose of this demonstration four features out of the list of available features were selected, including AVG_HA_USAGE, VAR_HA_USAGE, AGE, and EDUCATION_LEVEL. Accordingly, models for decision tree classification, logistic regression and SVM were created for all training sets. Moreover, decision tree classification was built using the default parameters, whereas SVM and logistic regression model parameters were tuned in order to boost their performance.

Afterward, predictions were made on the corresponding test datasets, while in the case of the model trained on the entire dataset, the prediction was also calculated on the whole dataset.

In Table 1, the performance of each algorithm is summarized. In the terms of correctly predicting dropout cases (TP), it can be seen that decision tree classification performed the best in all three cases (correctly predicting 62 out of 64, 49 out of 51 and 36 out of 37 dropouts cases in 50:50, 60:40 and 70:30 dataset splits respectively), while SVM performed the worst in all three cases (correctly predicting 57 out of 64, 40 out of 51 and 32 out of 37 dropout cases in 50:50, 60:40, and 70:30 dataset splits respectively). Furthermore, when it comes to correctly predicting non-dropouts (TN), decision tree classification again maintained the best overall performance, by having the highest performance in two out of three cases, namely 50:50 and 70:30 splits with 69 out of 70 and 38 out of 39 correctly predicted labels respectively. In the case of 60:40 dataset split, SVM outperformed decision tree classification by correctly predicting all cases (55) compared to 50 correctly predicted cases by decision tree classification.

**Figure 7:**
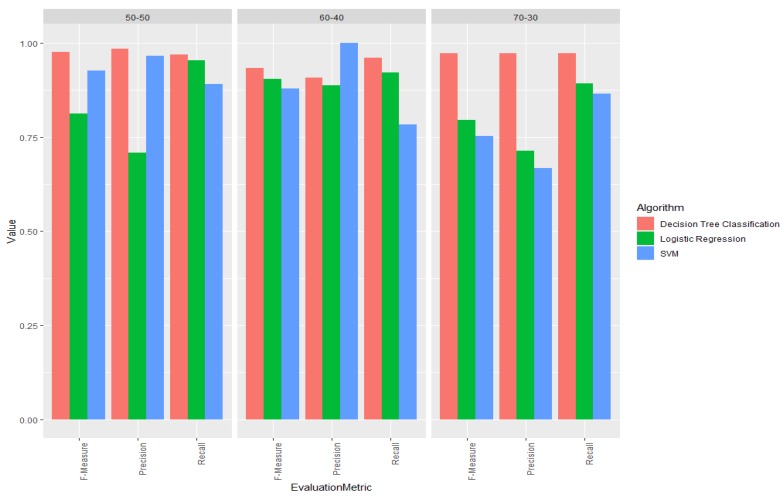
Comparison of precision, recall and f-mesure performance of different classification algorithms on varying training/test sizes.

Consequently, precision, recall, and f-measure results are shown in Figure 7. It can be observed, decision tree classification performed the best in all three cases in terms of recall and f-measure, as well as in two out of three cases in terms of precision. On the other hand, logistic regression and SVM algorithms had a comparable performance, which was in turn significantly lower than the performance of decision tree classification. This is especially the case in the third use case, where the dataset was divided according to the ratio 70:30. Performance drop can be potentially attributed to the fact that the further parameter tuning for those models is required. As a matter of fact, due to the ease of use and high performance, for this specific use case, decision tree classification is a recommended classification algorithm of choice.

By following the given use cases, a policy-maker can utilize the same algorithms on any combination of the available features, as well as perform the further parameter tuning in order to achieve the desired performance.

### 4.2. Utility Analysis: Survey

We have created a survey in order to inquire into the opinions of experts taking part in developing public health policies, regarding the application of new technologies and the platform, as far as the approach to hard-of-hearing persons is concerned. Policy-makers as target population were randomly selected from three different institutions. The survey was conducted at (a) the Croatian Institute of Public Health, whose experts propose measures to preserve and improve people’s health, (b) the Croatian Ministry of Health, that make decisions on the proposal of the Croatian Institute of Public Health, and (c) the Croatian Chamber of Health Professionals, which also cooperates with the Ministry of Health.

The research was conducted by the Institute of Public Health for the Osijek-Baranya County during April 2019. We used a questionnaire survey. Information about the EVOTION project was provided at the beginning of the questionnaire. The targeted population in the conducted research was a group of health policy-makers. We used purposive sampling methods. There were 38 respondents for the survey. The questionnaire consisted of 21 questions. Out of these questions, seventeen were related to the awareness of the respondents about the existence of the e-health strategy in Croatia, the availability of healthcare applications and the benefits they bring, as well as their views on the Platform. Basic demographic-related questions were included. The last four questions concerned the demographic traits, which included the gender composition of respondents, the age group (younger and older than 40 years old), the type of employment and the educational level. Questions from the MARS questionnaire for the evaluation of health mobile apps [19] and Third Global Survey on e-Health 2015 ((World Health Organization. 2015. Third Global Survey on eHealth. Available online: https://www.who.int/goe/survey/goe_2015_survey_en.pdf) were adopted and used. Also, some questions were used from an e-health questionnaire for the Czech Republic 2013 (E-Health Questionnaire for Czech Republic 2013. Available online: https://www.surveymonkey.com/r/LS2Q95L). The questionnaire included closed, multiple-choice and Likert-scale-styled questions [20].

#### 4.2.1. Statistical Analysis

The results are shown in contingency tables. Software SPSS (ver. 16.0, SPSS Inc., Chicago, IL, USA) was used for the analysis.

In total, 38 respondents took part in the survey, composing 17 (44.7%) men and 21 women (55.3%). Most of the respondents (55.3%) are younger than 40. Moreover, 97.4% of the respondents have a high degree of education (University study) (Table 2 and Table 3).

It was found that 42.1% of the respondents said that the national health coverage policy or strategy clearly refers to the usage of ICT or eHealth to support universal health coverage, 26.3% of respondents think that this policy is unclear, and 31.6% of respondents do not know. Further, 63.2% said that the country has a national eHealth policy or strategy, 10.5% of the respondents said it does not, and 26.3% of the respondents do not know. When asked “Does your national eHealth policy or strategy refer to the objectives of universal health coverage or its key elements?” (such as access, quality, and cost of care), 52.6% said that they do not know, 34.2% said that the national eHealth policy or strategy refers to the universal health coverage objectives or its key elements, and 13.2% of the respondents said that the policy or the strategy do not refer to these objectives. In addition, 65.8% of the respondents said that the country has a national health information system policy or strategy, whereas 34.2% said that it does not. Further, 78.9% of respondents are aware of the availability of health apps for smartphones. All respondents are aware of the harmful impact that noise can have on health. Moreover, 97.4% of the respondents said that the platform was useful, 71.1% of the respondents said that this approach is successful in hearing loss prevention and management strategy, and 28.9% said they do not know (Table 4).

Respondents believe that primary measurable objectives of an e-health system should be: cost saving (78.9%), quality control (76.3%), improvement of health outcomes (63.2%), development of new products and services (36.8%), and easier patient access to care (5.3%) (Table 5).

Most of the respondents download social apps (50%), health and lifestyle (43.2%), education apps (37.8%), then news (34.2%), business apps (31.6%), games & books app (23.7%), and other (Table 6).

**Figure 8:**
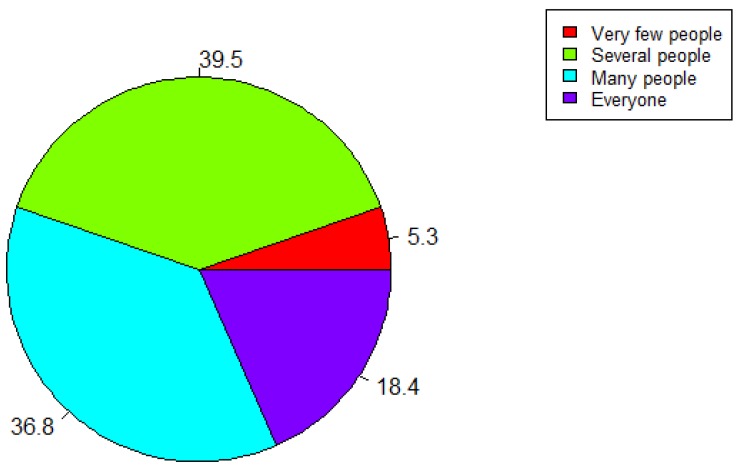
“Would you recommend this platform to people who might benefit from it?”.

As can be observed from the Figure 8, most of the respondents (39.5%) said they would recommend the platform to some people, and 36.8% of the respondents believe there are many people to whom they would recommend the platform. Moreover, 18.4% of the respondents would recommend such a platform to everyone, and 5.3% would not recommend it to anyone.

**Figure 9:**
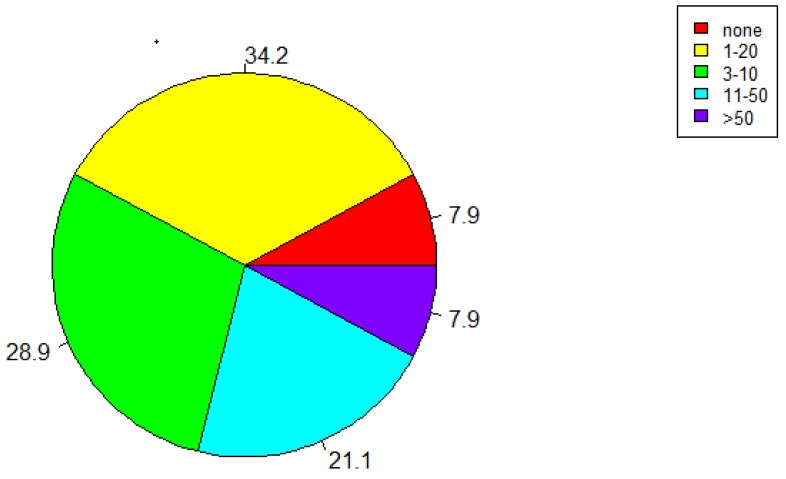
“How many times do you think you would use this platform in the next 12 months if it was relevant to you?”

In the next year, 34.2% of the respondents would use the app 1-2 times, 28.9% of the respondents would use the platform 3–10 times, 21.1% respondents would use the platform 11–50 times, and the percentage of respondents who would not use the platform at all is the same to the percentage of respondents who would use it more than 50 times (7.9%) (Figure 9).

It was found that 31.6% of the respondents think that e-health improves the quality of service, 47.4% had a neutral stance toward the statement, whereas 18.4% of the respondents do not agree and 2.6% strongly disagree with the statement. Further, 26.3% of the respondents think that e-health improves health care, 50% are neutral, 18.4% think it does not improve health care, and 5.3% of the respondents strongly disagree with the statement. Moreover, 52.6% of the respondents agree with the statement that e-health is important for creating new health care products and services, 31.6% strongly agree, and 15.8% are neutral (Table 7).

**Figure 10:**
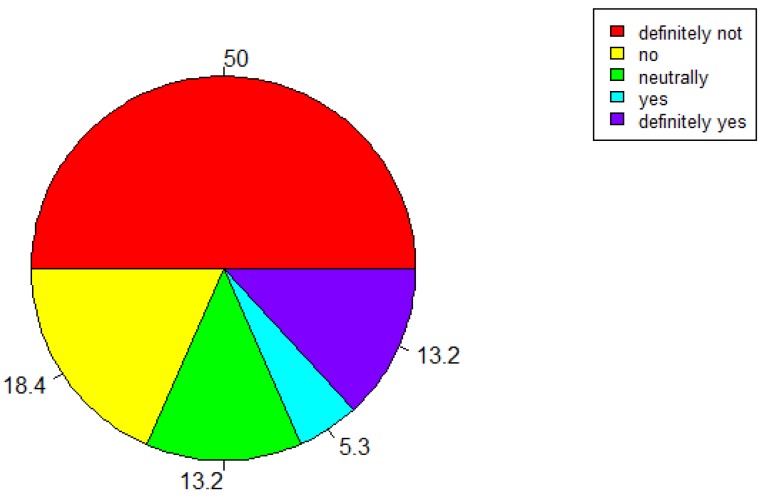
“Would you pay for our platform?”

As can be observed from the Figure 10, 50% of the respondents said they would not pay to use the app and 18.4% would not pay. The percentage of respondents who would pay is the same to the percentage of respondents who are neutral (13.2%), whereas 5.3% said they would pay to use the app (Figure 10).

#### 4.2.2. Discussion on Survey Results

As far as the ICT is concerned, 42.1% of the respondents said that the national health coverage policy or strategy clearly refers to the usage of ICT or eHealth to support universal health coverage. Due to the percentage of respondents who think that the policy is unclear (26.3%) and those who do not know the answer (31.6%), the question arises as to how well the respondents are informed about and familiar with the national policy and health insurance strategy, as well as how aware they are of the connection between this strategy with ICT and e-health. Even though informatization and the introduction of e-health have been done for the past 10 years (Strategic plans of the Ministry of Health of the Republic of Croatia. (n. d.). Available online: https://zdravlje.gov.hr/pristup-informacijama/strategije-planovi-i-izvjesca/strateski-planovi/2672), 10.5% of the respondents said the country does not have a national e-health policy or strategy, and 26.3% does not know whether such a policy or strategy exists. Further, 52.6% of the respondents do not know and 13.2% of the respondents think that the national e-health policy or strategy does not refer to the objectives of the universal health coverage or its key elements (e.g., access, quality, cost of care). More than a third of the respondents (34.2%) think that the country does not have a national health information system policy or strategy. The acquired data are concerning if we take into account that policy-makers from three key institutions participated in the survey (Croatian Institute of Public Health, Croatian Ministry of Health and the Croatian Chamber of Health Professionals). This might be the result of a lack of human resources policies focusing on expertise and competences, while insufficient staff education is prevalent. Further research should certainly clarify this issue. In Lavis’s study [2], over a third of researchers did not feel that their country’s health research environment was supportive of individuals undertaking knowledge translation activities (37%) and nearly half (47%) did not feel that there were sufficient structures and processes in place to link researchers and their target audience. The respondents from our survey think that the primary measurable objectives of an e-health system should be: cost-saving, quality control, improvement of health outcomes, development of new products and services and easier patient access to care. One third of respondents think that e-health improves the quality of service. Only 26.3% of respondents think that health care is improved by e-health. More than half of the respondents recognize the potential of e-health in developing new products and services in health. Similar to our results, other studies found that researchers may feel that their work is not utilized because of competing interests, lack of funding, or a lack of will on the decision-makers’ part [21]. Other studies also identified numerous barriers to the use of evidence to inform policy such as political governance, the bureaucratic process and an overall lack of trust within the system [2].

Most of the respondents (97.4%) involved in our survey think that the platform is useful, and all of them are aware of the harmful impact that noise can have on health. Even though almost all the respondents have recognized the usefulness of the platform, 39.5% would recommend the platform to some people, and 5.3% would not recommend it to anyone. Respondents’ personal views might be the reason for such results. In this context, 71.1% of the respondents said that this approach (EVOTION) is successful in hearing loss prevention and as a management strategy. Despite recognizing the usefulness of the platform, more than half of the respondents (58.4%) would not pay to use the EVOTION app, whereas only 5.3% would pay to use it. Although respondents did acknowledge the usefulness of the platform, only 7.9% of them would use it more than 50 times in a year. Evidence only plays one role (and unfortunately it is sometimes a small one) in the policymaking process and external factors as well as political and institutional forces usually have a stronger role in the policymaking process [3]. In conclusion, it is necessary to intensify the education of experts working with health policies and it is also suggested to develop lifelong learning curriculums incorporating e-health for policy-makers.

## 5. Conclusions

In this paper, we present a data-informed policymaking platform. To show the potentiality, utility, and usability of the platform to the policy-makers, we introduce a scenario based on HL of the EVOTION project and then present a walkthrough example of how the policy-makers can utilize machine learning and statistical capabilities of the platform in order to design a policy aimed at lower dropout from the HL EVOTION trial. Concluding, we investigated the views of health system policy-makers involved in the policy development process in Croatia regarding the use of our platform. We presented the extensive results of a survey conducted on policymaking experts.

## Figures and Tables

**Table 1:** Performance evaluation of the different classification algorithms on varying training/test sizes.

Algorithm	50:50				60:40				70:30			
	TP	FP	TN	FN	TP	FP	TN	FN	TP	FP	TN	FN
SVM	57	2	68	7	40	0	55	11	32	16	23	5
Logistic Regression	61	25	45	3	47	6	49	4	33	13	26	4
Decision Tree Classification	62	1	69	2	49	5	50	2	36	1	38	1

**Table 2:** “Respondents by sex and age group”.

Age	M	F	All
	N (%)	N (%)	N (%)
<40	9 (52.9)	12 (57.1)	21 (55.3)
≥40	8 (47.1)	9 (42.9)	17 (44.7)
all	17 (100)	21 (100)	38 (100)

**Table 3:** “Choose the highest level of your education”.

Level of Education	M	F	All
	N (%)	N (%)	N (%)
undergraduate study	1 (5.9)	0	1 (2.6)
graduate study	16 (94.1)	21 (100)	37 (97.4)
all	17 (100)	21 (100)	38 (100)

**Table 4:** What should be the primary measurable objectives of an e-health system? (you can answer more than one).

Question	Yes	No	Don’t Know	All
	N (%)	N (%)	N (%)	N (%)
Does your national universal health coverage policy or strategy clearly refer to the use of ICT or eHealth to support universal health coverage?	16 (42.1)	10 (26.3)	12 (31.6)	38 (100)
Does your country have a national eHealth policy or strategy?	24 (63.2)	4 (10.5)	10 (26.3)	38 (100)
Does your national eHealth policy or strategy refer to the objectives of universal health coverage or its key elements? (such as access, quality and cost of care)	13 (34.2)	5 (13.2)	20 (52.6)	38 (100)
Does your country have a national health information system (HIS) policy or strategy?	25 (65.8)	13 (34.2)		38 (100)
Are you aware of the availability of health apps (applications) for smartphones?	30 (78.9)	8 (21.1)	-	38 (100)
Are you aware that noise can have a harmful impact on health?	38 (100)	-	-	38 (100)
Do you find this Platform usefull?	37 (97.4)	1 (2.6)	-	38 (100)
Do you feel that this approach is more successful in prevention and the management of hearing loss?	27 (71.1)	-	11 (28.9)	38 (100)

**Table 5:** What should be the primary measurable objectives of an e-health system? (you can answer more than one).

Objective	Yes	No	All
	N (%)	N (%)	N (%)
cost saving	30 (78.9)	8 (21.1)	38 (100)
quality control	29 (76.3)	9 (23.7)	38 (100)
improvment of health outcomes	24 (63.2)	14 (36.8)	38 (100)
development of new products and services	14 (36.8)	24 (63.2)	38 (100)
easier patient access to care	2 (5.3)	36 (94.7)	38 (100)
all	17 (100)	21 (100)	38 (100)

**Table 6:** What type of applications do you download?

Application Type	Yes	No	All
	N (%)	N (%)	N (%)
games	9 (23.7)	29 (76.3)	38 (100)
education	14 (37.8)	23 (62.2)	37 (100)
books	9 (23.7)	29 (76.3)	38 (100)
news	13 (34.2)	25 (65.8)	38 (100)
social	19 (50.0)	19 (50.0)	38(100)
health and lifestyle	16 (43.2)	21 (56.8)	37 (100)
business	12 (31.6)	26 (68.4)	38 (100)
other	7 (18.9)	30 (81.1)	37 (100)
all	17 (100)	21 (100)	38 (100)

**Table 7:** Please tick the box that best describes what you think “e-health improve quality of service“.

Opinion	Strongly Agree	Agree	Neutral	Disagree	Strongly Disagree	All
	N (%)	N (%)	N (%)	N (%)	N (%)	N (%)
Please tick the box that best describes what you think. „e-health improve quality of service“	12 (31.6)	18 (47.4)	7 (18.4)	1 (2.6)	-	38 (100)
„e-health solutions improve health care outcames“	10 (26.3)	19 (50)	7 (18.4)	1 (2.6)	-	
„e-health is important for creating new health care products and services?“	12 (31.6)	20 (52.6)	6 (15.8)	-	-	
